# A Stationary Wavelet Transform Based Approach to Registration of Planning CT and Setup Cone beam-CT Images in Radiotherapy

**DOI:** 10.1007/s10916-014-0040-2

**Published:** 2014-04-13

**Authors:** Jun-min Deng, Hai-zhen Yue, Zhi-zheng Zhuo, Hua-gang Yan, Di Liu, Hai-yun Li

**Affiliations:** School of biomedical engineering, Capital Medical University, Beijing, China 100069

**Keywords:** Image registration, Planning CT, Cone beam-CT, Stationary wavelet transform, Mutual information

## Abstract

Image registration between planning CT images and cone beam-CT (CBCT) images is one of the key technologies of image guided radiotherapy (IGRT). Current image registration methods fall roughly into two categories: geometric features-based and image grayscale-based. Mutual information (MI) based registration, which belongs to the latter category, has been widely applied to multi-modal and mono-modal image registration. However, the standard mutual information method only focuses on the image intensity information and overlooks spatial information, leading to the instability of intensity interpolation. Due to its use of positional information, wavelet transform has been applied to image registration recently. In this study, we proposed an approach to setup CT and cone beam-CT (CBCT) image registration in radiotherapy based on the combination of mutual information (MI) and stationary wavelet transform (SWT). Firstly, SWT was applied to generate gradient images and low frequency components produced in various levels of image decomposition were eliminated. Then inverse SWT was performed on the remaining frequency components. Lastly, the rigid registration of gradient images and original images was implemented using a weighting function with the normalized mutual information (NMI) being the similarity measure, which compensates for the lack of spatial information in mutual information based image registration. Our experiment results showed that the proposed method was highly accurate and robust, and indicated a significant clinical potential in improving the accuracy of target localization in image guided radiotherapy (IGRT).

## Background

To achieve the best therapeutic outcome, modern radiotherapy has attempted a variety of ways to maximize the damage to the tumor while sparing surrounding normal tissues [1, 3–5]. The accurate targeting of tumor has been playing an important role in the implementation of successful radiotherapy, which introduced the concept of image guided radiotherapy (IGRT). As one of the key steps in targeting tumor, the registration between planning CT images and CBCT (Cone beam-CT) images has been widely explored and its techniques have been improved significantly since the advent of CBCT.

Image registration techniques generally rely on the information of the images themselves. Current image registration methods fall roughly into two categories: geometric features-based and image grayscale-based [2]. To date, these image registration methods have been widely used to perform the registration between planning CT and CBCT images. Based on the deformation with intensity simultaneously corrected, a CT to CBCT deformable registration approach was proved to be robust against the CBCT artifacts and intensity inconsistency [3]. Free-form deformable registration algorithm, which resulted in a high correlation between CBCT and the new planning CT, was also successfully conducted [4]. Multiscale registration, which decomposed the registering images into a series of scales and registered the coarser scales of the images iteratively, was regarded as an effective method for the registration between CT and daily CBCT images [5]. Registration techniques based on mutual information (MI) belong to the image grayscale-based registration method and have been widely applied to multi-modal and mono-modal image registration tasks. A multi-modal retinal image registration, which was based on improved mutual information using adaptive probability density estimation, resulted in high accuracy and efficiency [6]. Three–dimensional registration techniques based on mutual information could be also applied to the alignment of brain tissues in magnetic resonance imaging time-series or PET [7, 8]. A comparison between standard mutual information and normalized mutual information indicated that normalized mutual information is more stable and robust in that it is immune to the variation of entropy [9]. The application of mutual information is a very effective strategy for image registration, but the traditional mutual information method only focuses on the image intensity information, with spatial information neglected, which leads to the instability to intensity interpolation [10]. With regard to registration of medical images, spatial information is very important and should be incorporated into grayscale-based based registration algorithms. A 3D-2D registration of CT and X-ray images incorporated the spatial information in a variational approximation and obtained a high registration accuracy [11]. Positions with large gradient usually correspond to tissue transition, which provides spatial information [12]. Therefore, wavelet transform was recently applied to image registration [13, 14]. Daubechies complex wavelet transform, which is shift invariant and provides phase information, was successfully used to achieve the fusion of multimodal medical images [15]. A flexible multiscale and shift-invariant representation of registered images was firstly obtained by using stationary wavelet transform, and then the registration through pulse-coupled neural network was performed on the new representation [16, 17].

Some studies incorporated the gradient information of medical images in the mutual information to compensate for the lack of spatial information [18, 19]. These methods produced gradient images using the sum of the squares of the corresponding sub-band coefficients. However, gradient images generated this way lack diagonal components, leading to the loss of edge information and low registration accuracy. In registrations between planning CT images and CBCT images, the noise in CBCT images usually results in poor resolution in low contrast areas of the images and blurs edges of images.

In this paper, we proposed a registration method based on stationary wavelet transform (SWT) with translational invariance. The translational invariance of the stationary wavelet is conducive to highlighting edge features of an image and improves the registration accuracy. Experiments showed that our algorithm is robust.

## Materials and methods

### Materials

For planning CT images and setup CBCT images in radiotherapy, Siemens large aperture CT and Varian Rapid Arc CBCT are used for image registration. The image parameters for CT are as follows: image matrix 512 × 512; pixels size 1.17 × 1.17 mm; the image parameters for CBCT are as follows: image matrix 384 × 384; pixel size 1.27 × 1.27 mm. All the participants gave their informed consent and the Ethics Committee of Beijing Xuanwu Hospital Affiliated to Capital Medical University approved the protocol of this study.

### Methods

In our proposed method, the reference image and floating images were decomposed with three levels using stationary wavelet transform. Low-frequency components of wavelet produced in all levels during the decomposition were set to zero, and a gradient image of the original image was obtained by performing inverse wavelet transform on remaining high-frequency components. Then, the mutual information of the original image and the gradient image was calculated by using the normalized mutual information as the similarity measure. Finally, a new similarity measure was synthesized with a weighting function. The Powell algorithm was used for multi-parameter optimization to produce the final spatial transformation parameters for the image registration.

### Stationary wavelet transform of image

**Fig. 1:**
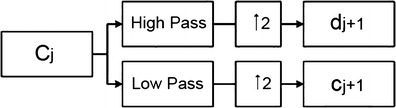
Schematic diagram of the jth level SWT decomposition. The signal *C*_*j*_ is decomposed into low frequency components *c*_*j* + 1_ and high frequency components *d*_*j* + 1_ corresponding to the high pass and low pass filters, respectively

Nason and Silverman introduced the stationary wavelet transform in 1995 [20]. In contrast to orthogonal wavelets, stationary wavelet, also known as non-sampling wavelet transform, has the properties of redundancy, translational invariance, capability of providing more approximate estimation of continuous wavelet transform. As an effective mathematical tool for edge detection [21–24], its advantages include the local time-frequency characteristics and multi-resolution analysis capability of wavelet transform. The *j*th-level decomposition of SWT is shown in Fig. 1.

The decomposition formulas of SWT are as follows:

1 $$ \begin{array}{l}{A}_{j,{k}_1,{k}_2}=\sum_{n_1}\sum_{n_2}{h}_0^{\uparrow {2}^j}\left({n}_1-2{k}_1\right){h}_0^{\uparrow {2}^j}\left({n}_2-2{k}_2\right){A}_{j-1,{n}_1,{n}_2}\hfill \\ {}{D}_{j,{k}_1,{k}_2}^1=\sum_{n_1}\sum_{n_2}{h}_0^{\uparrow {2}^j}\left({n}_1-2{k}_1\right){g}_0^{\uparrow {2}^j}\left({n}_2-2{k}_2\right){A}_{j-1,{n}_1,{n}_2}\hfill \\ {}{D}_{j,{k}_1,{k}_2}^2=\sum_{n_1}\sum_{n_2}{g}_0^{\uparrow {2}^j}\left({n}_1-2{k}_1\right){h}_0^{\uparrow {2}^j}\left({n}_2-2{k}_2\right){A}_{j-1,{n}_1,{n}_2}\hfill \\ {}{D}_{j,{k}_1,{k}_2}^3=\sum_{n_1}\sum_{n_2}{g}_0^{\uparrow {2}^j}\left({n}_1-2{k}_1\right){g}_0^{\uparrow {2}^j}\left({n}_2-2{k}_2\right){A}_{j-1,{n}_1,{n}_2}\hfill \end{array} $$

where ${A}_{j,{k}_1,{k}_2}$, ${D}_{j,{k}_1,{k}_2}^1$, ${D}_{j,{k}_1,{k}_2}^2$, ${D}_{j,{k}_1,{k}_2}^3$ are the low frequency components (LL), the horizontal high-frequency component (LH), vertical high-frequency component (HL) and diagonal components (HH) of the stationary wavelet transform, respectively. ${h}_0^{\uparrow {2}^j}$ and ${g}_0^{\uparrow {2}^j}$ are used to denote that 2^j^-1 zeros are inserted between the two points *h*_0_ and *g*_0_. The corresponding reconstruction algorithm (IDSWT) is

2 $$ \begin{array}{l}{A}_{j-1,{n}_1,{n}_2}=\frac{1}{4}{\displaystyle \sum_{i=0}^3\left\{{\displaystyle \sum_{k1}{\displaystyle \sum_{k2}{h}_1\left({n}_1-2{k}_1-i\right){h}_1\left({n}_2-2{k}_2-i\right){A}_{j,{k}_1,{k}_2}}}\right.}\hfill \\ {}\kern6em +{\displaystyle \sum_{k_1}{\displaystyle \sum_{k_2}{h}_1\left({n}_1-2{k}_1-i\right){g}_1\left({n}_2-2{k}_2-i\right){D}_{j,{k}_1,{k}_2}^1}}\hfill \\ {}\kern6em +{\displaystyle \sum_{k_1}{\displaystyle \sum_{k_2}{g}_1\left({n}_1-2{k}_1-i\right){h}_1\left({n}_2-2{k}_2-i\right){D}_{j,{k}_1,{k}_2}^2}}\hfill \\ {}\kern6em \left.+{\displaystyle \sum_{k_1}{\displaystyle \sum_{k_2}{g}_1\left({n}_1-2{k}_1-i\right){g}_1\left({n}_2-2{k}_2-i\right){D}_{j,{k}_1,{k}_2}^3}}\right\}\hfill \end{array} $$

**Fig. 2:**
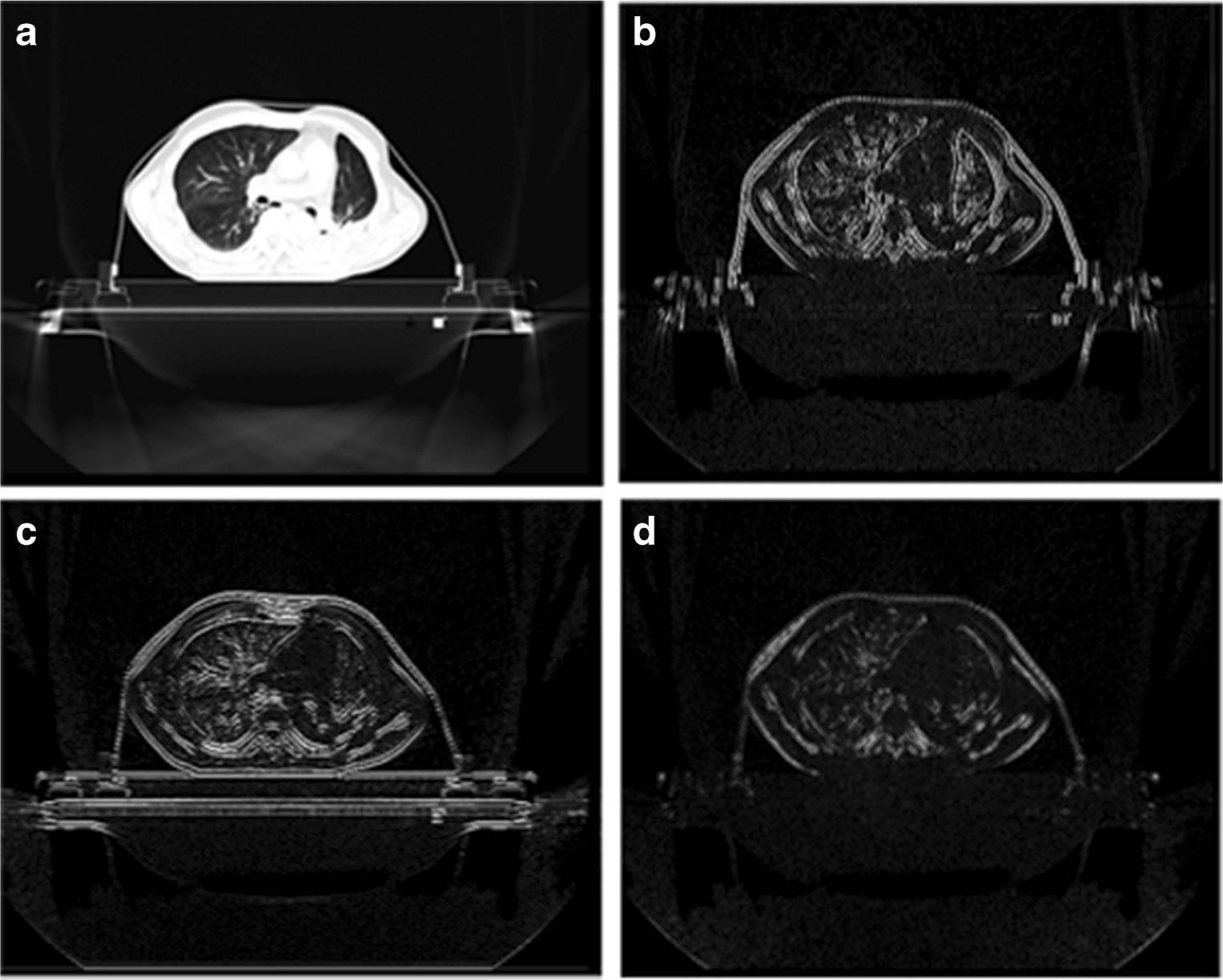
The images produced after the decomposition with SWT. Parts **a**, **b**, **c**, **d** are the low frequency components, the horizontal high frequency components, the vertical high-frequency components and diagonal components of the stationary wavelet transform, respectively

The components of the image after the SWT are shown in Fig. 2.

### Synthesized gradient image based on stationary wavelet transform

**Fig. 3:**
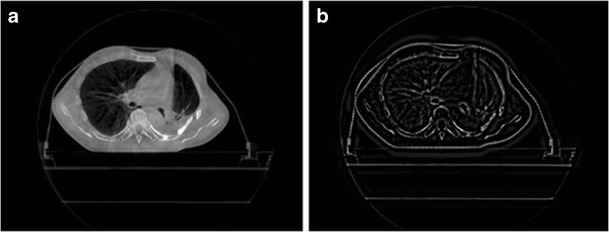
The CBCT image and the gradient image produced after the SWT. Part a is the original CBCT image and Part b is the gradient image after the SWT

The high-frequency sub-band of wavelet transform has the ability to highlight the differences between neighboring pixels in an image [25]. Large wavelet coefficient indicates the boundary of two distinct intensity regions in the original image. Stationary wavelet transform is translationally invariant, which helps to identify the image edge features. In order to improve the resolution of edge details, image with prominent edge features can be reconstructed by using the inverse SWT with the three groups of wavelet vectors (LH, HL, HH). The CBCT image and the gradient image generated with SWT are shown in Fig. 3.

### Similarity measure

In this paper, mutual information is used as the similarity measure. As a basic concept of information theory, mutual information is generally used to describe the statistical correlation between two systems, or the amount of information of a system contained in another system. In multimodality image registration, when the spatial positions of two images are completely consistent, the mutual information, i.e., the information of one image expressed by another image, is maximum. The mutual information can be expressed by entropy which describes the complexity or uncertainty of a system. The entropy of the system A is defined as

3 $$ H(A)=-{\displaystyle \sum_a{p}_A(a) \log {p}_A(a)} $$

The joint entropy of two systems is defined as

4 $$ H\left(A,B\right)=-{\displaystyle \sum_{a,b}{p}_{A,B}\left(a,b\right) \log {p}_{A,B}\left(a,b\right)}. $$

where *a* ∈ *A*, *b* ∈ *B*, and *p*_*A*_(*a*) is the marginal probability density function, *p*_*A*,*B*_(*a*, *b*) is the joint probability density function. The mutual information between the two systems is thus expressed as

5 $$ I\left(A,B\right)=H(A)+H(B)-H\left(A,B\right). $$

Mutual information is sensitive to the amount of overlap between images and normalized mutual information (NMI) has been proposed to overcome this problem. It is defined as

6 $$ NMI\left(A,B\right)=\frac{H(A)+H(B)}{H\left(A,B\right)}. $$

### Registration method based on stationary wavelet transform

We denoted the normalized mutual information of the original image as NMIi and that of the synthesized gradient image based on the SWT as NMIg. They were calculated using Eq. (1). Jiangang Liu merged original images with gradient information using the method described below, and the new similarity measure was given by [24]:

7 $$ NMI=f\left(v\left( NM{I}_i, NM{I}_g\right)\right) NM{I}_i+\left(1-f\left(v\left( NM{I}_i, NM{I}_g\right)\right)\right) NM{I}_g $$

where

8 $$ f(v)=1/\left(1+ \exp \left(-\left(v-0.5\right)/T\right)\right) $$

9 $$ v\left(x,y\right)=\left(x+y\right)/2 $$

**Fig. 4:**
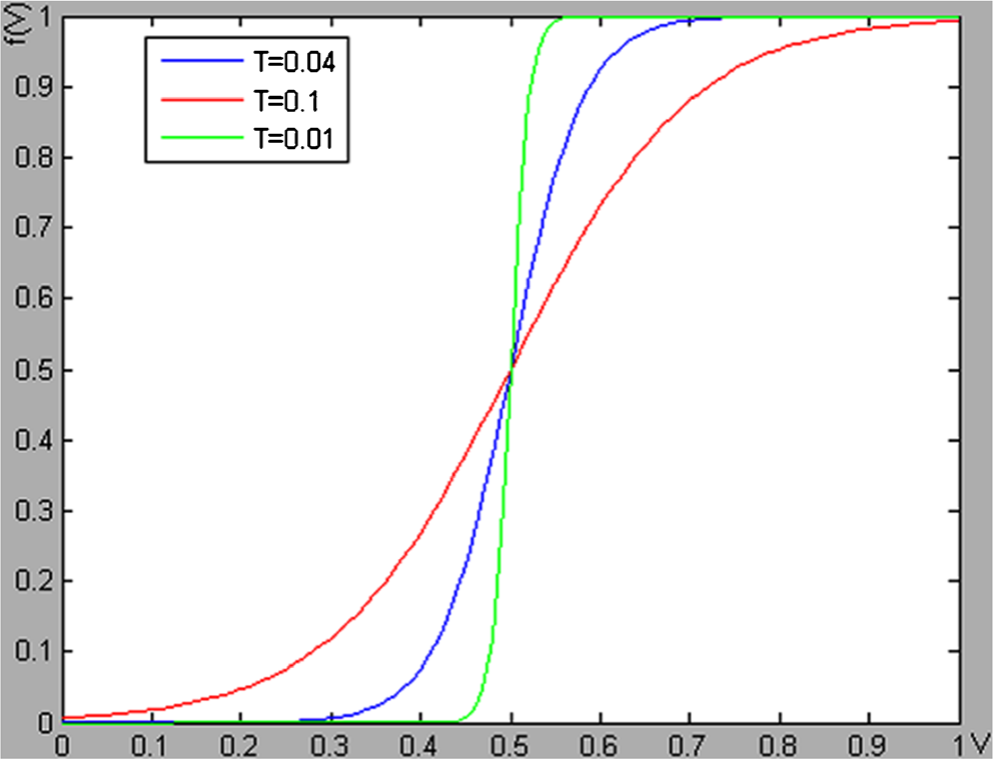
Weighting function *f*(*v*). The shapes of the weighting function depends on the parameter T, the blue line, the red line and the green line are corresponding to the T values of 0.04,0.1 and 0.001, respectively

The weighting function f(v) is shown in Fig. 4, where T is a time constant used to control the shape of f(v). The weighting function is essentially a logistic function, which has the properties of saturation, differentiability and nonlinearity. It also has a maximum and a minimum. f(v) is an ideal weighting function for merging registration function. According to our experience, T = 0.04 is an appropriate value.

Our method follows this procedure:

- Step 1: decompose the reference image R and floating image F respectively using stationary wavelet transform;
- Step 2: assign the coefficients of low-frequency in all levels of stationary wavelet decomposition to zero and perform inverse transform to reconstruct corresponding gradient images;
- Step 3: combine the original image and the synthesized gradient image using the weighting function to form a new similarity measure;
- Step 4: use the Powell multi-parameter optimization algorithm to optimize the space transformation parameters (*Δx*, *Δy*, *θ*) and the registration is completed.

### Optimization algorithm

Two-dimensional image registration is essentially a multi-parameter optimization problem, namely, searching three optimal registration parameters (two translational parameters and a rotational parameter) to maximize the mutual information. In this paper, the Powell multi-parameter optimization algorithm and the Brent one-dimensional search algorithm are used to optimize the parameters.

## Results

Our algorithm was implemented in Matlab R2008a. We selected ten medical images as the reference images, and the floating images were generated with spatial transformation of the corresponding reference images. As the preset transformation parameters in X and Y directions and the rotation angle *θ* (As shown in Table 1) were known, judgment of the correctness and registration accuracy of the algorithm was straightforward. The smaller the gray level difference between the image F after registration and the reference image R is, the higher the registration accuracy is.

**Table 1:** Experiment results and error

Image	Preset transformation parameters (*Δx*, *Δy*, *θ*)	Our proposed method (*Δx*, *Δy*, *θ*)	The mutual information method (*Δx*, *Δy*, *θ*)	MSE of pre-registration	MSE1	MSE2
1	10 15 −3	10 15 −3	10.04 14.96 −2.99	29.2475	0	0.8284
2	10 10 −3	10 10 −3	10.05 9.96 −2.88	29.0496	0	5.4321
3	20 15 −3	19.95 15.04 −3.01	20 15 −3	32.5702	0.5904	0
4	20 −10 −5	20 −10 −5	20 −10 −5	38.3434	0	0
5	18 −8 −3.45	18 −8 −3.45	18. −8 −3.45	36.2414	0	0
6	30 20 −3	30 19.99 −3	31.63 19.32 −3	32.2632	0.07	8.2306
7	10 25 −3	10 25 −3	10 25 −3	31.3465	0	0
8	10 −16 −3	10 −16 −3	10.02 −16.01 −3	35.8125	0	0.1496
9	5 8 2	5 8 2	5 8 2	28.4468	0	0
10	10 12 2	10 12 2	10 12 2	30.8140	0	0

*MSE*1 registration error of the proposed method; *MSE*2 registration error of the mutual information method;

*Δx*, *Δy* are transformation parameters along X and Y directions, both measured in mm;

*θ* is the rotation angle of the transformation, measured in rad

Root mean square error (MSE), which is defined as follows, is employed as the registration error [26–29].

10 $$ MSE=\sqrt{\frac{1}{ MN}{\displaystyle \sum_{i-1}^M{\displaystyle \sum_{j-1}^N{\left[R\left(i,j\right)-F\left(i,j\right)\right]}^2}}}. $$

The smaller the values of MSE are, the higher the registration accuracy is. If two images are identical, the MSE = 0. We took ten images with preset transformation parameters as the experiment data, the experiment results of which are shown in Table 1.

Judged from the above experiment results, the proposed registration method is accurate (on the order of subpixel) and robust. However, compared with mutual information registration, the proposed method is time-consuming, because it needs to calculate not only the mutual information between the original images, but also the mutual information with the gradient images, which increases the computation load.

The CBCT image is shown in Fig. 3a. Because of smaller image matrix and larger pixel size, CBCT images must be up-sampled to the size of the CT image before the registration. Due to a considerable difference between our CT and CBCT images, the initially linear shifting (80 and 80 mm along X and Y directions, respectively) of the CBCT images was performed to manually narrow the difference for the reduction of latter registration time. In order to test the CT and CBCT images registration algorithm, we generated ten images by performing manual spatial transformation to CBCT images in advance (as shown in Table 2). Among the ten images, five images were transformed in Y direction linearly, and other five images were transformed linearly in X direction. They were then registered with the corresponding layer of CT images. If the corresponding translation term of the registration is linear, and the rotation angle is close to 0°, high registration accuracy is indicated. The registration results are shown in Table 2.

**Table 2:** Registration results of the CT and CBCT images

Image	Preprocessing parameters (*Δx*, *Δy*, *θ*)	Results of proposed method (*Δx*, *Δy*, *θ*)	Results of mutual information method (*Δx*, *Δy*, *θ*)
1	80 80 0	−3.28 −46.22 −0.30	−2.5000 −45.78 −0.15
2	80 85 0	−1.89 −41.29 −0.04	−17.18 −27.90 −3.42
3	80 90 0	−2.13 −36.25 −0.05	−2.00 −36.34 −0.03
4	80 95 0	−1.71 −31.43 −0.03	−3.73 −29.93 −0.32
5	80 100 0	−2.33 −26.11 −0.09	−2.36 −25.91 −0.11
6	85 80 0	2.46 −46.25 −0.06	2.33 −45.77 −0.16
7	90 80 0	7.26 −46.38 −0.04	8.40 −46.59 0.02
8	95 80 0	12.53 −45.93 −0.11	12.61 −46.09 −0.10
9	100 80 0	18.39 −46.49 −0.01	17.82 −46.22 −0.06
10	105 80 0	23.58 −46.76 0.05	22.83 −46.22 −0.06

*Δx*, *Δy* are transformation parameters along X and Y directions, and the corresponding units are mm and mm;

*θ* is the rotation angle of the transformation, and the corresponding unit is rad

**Fig. 5:**
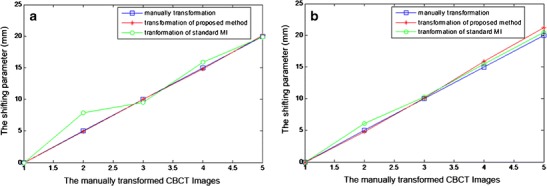
The linearity of transformation parameters in X and Y directions. Part a shows the transformation in Y direction and part b shows the transformation in X direction

In order to compare our proposed method with standard mutual information, the linearity of the transforming variation along X and Y directions was represented in Fig. 5

**Fig. 6:**
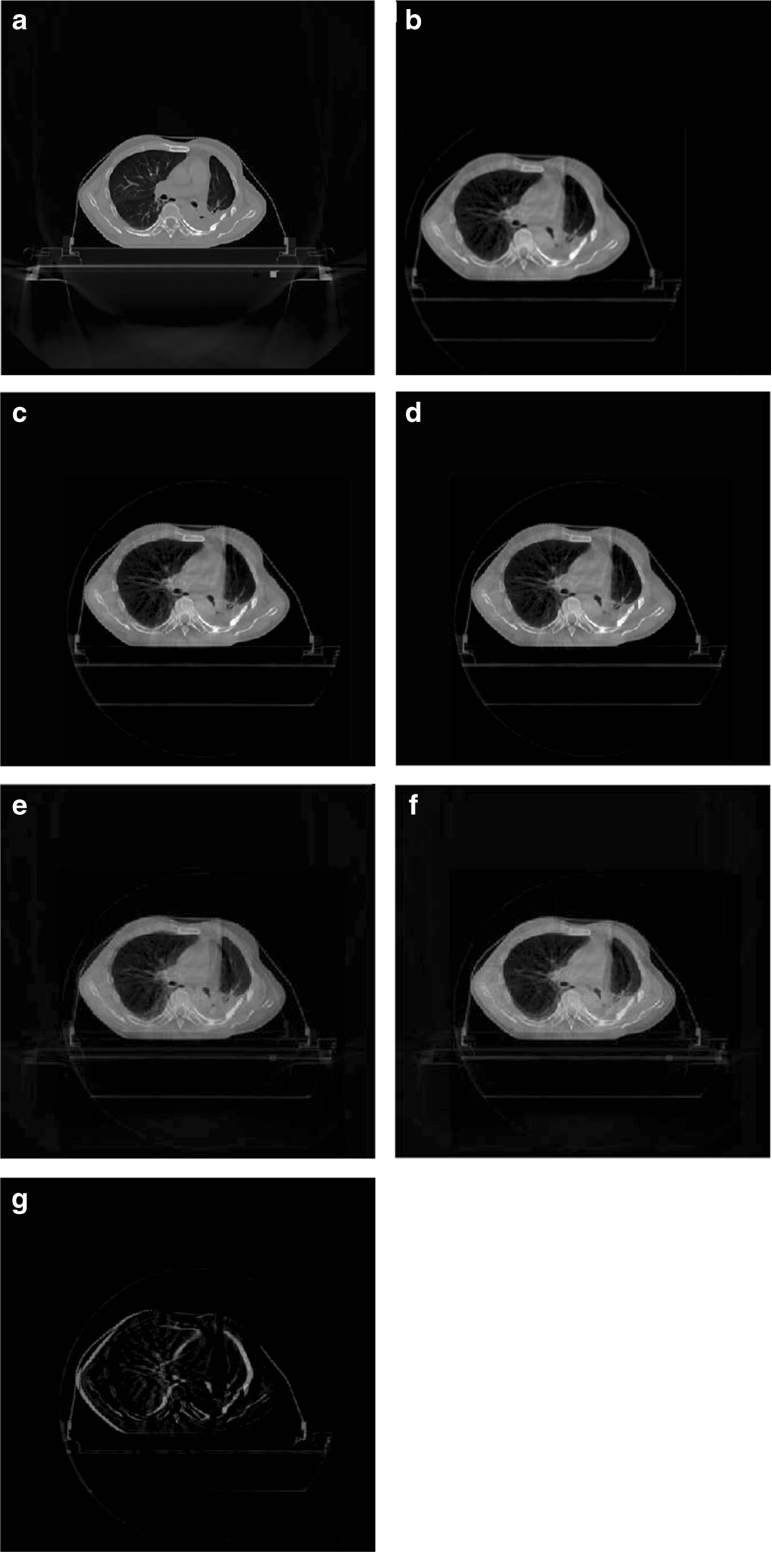
Results of registering CBCT to CT images. Part **a** is the original CT image; Part **b** is the CBCT image; Part **c** is the registration result by using conventional MI method; Part **d** is the .registration result by using our proposed method; Part **e** is the fusion of CBCT and CT images with conventional MI method; Part **f** is the fusion of CBCT and CT images with our proposed method; Part **g** is the difference (**f**–**e**) of registered CBCT images by using the two methods

As can be seen from Table 2 and Fig. 5, with our proposed method, the translations in the Y direction in the first five images, and in the X direction in the last five images appear linear. Therefore, the proposed method is more robust than the traditional mutual information registration. The registration results of CBCT to CT images are shown in Fig. 6.

## Discussion

Based on mutual information and stationary wavelets transform, the registration of CT and manually transformed CT images resulted in a lower MMSE compared with standard mutual information, which indicated that the algorithm we proposed was more accurate. The registration results of CT and CBCT images showed that the transformation parameters of our registration method was more linearly related to the preprocessing parameters along the corresponding directions, which indicated that our method was also more robust. The stationary wavelet transform can be applied to obtain the spatial information of the registered images and the normalized mutual information can be used as the similarity measure in the registration. The combination of the above techniques yielded an effective registration of CT and CBCT, which is indispensable for the accurate location of tumor in radiotherapy. In our future investigation, we will focus on improving the speed of the algorithm. In addition, compared with other methods, the proposed registration algorithm calculated the mutual information of the original image and the gradient image respectively, which increases the computational cost. Shortening the algorithm running time will be the focus of our further research.

There are still some limits in our work. Firstly, we just tried to use the gradient information to investigate the effects of registration between CT and CBCT images, so compared with other state-of-art method performing on multimodal image registration (such as registration of CT, MRI, PET images, etc. ) [30–34], our proposed method may not perform so well. In our future work, we will continue to improve our method and apply it on multimodal images for a further evolution.

## Conclusions

In this paper, we proposed a medical image registration algorithm based on SWT and mutual information. The algorithm synthesizes a gradient image based on the translational invariance of SWT, and incorporates it into the mutual information calculation of the original image by the weighting function to obtain a new similarity measure. The proposed method effectively overcomes the weakness of mutual information registration for the lack of spatial information. Experiment results showed that the proposed method is robust and accurate. As for the registration between planning CT images and setup CBCT images in radiotherapy, SWT is data redundanct and translationally invariant, which is conducive to identify sharp variations in the image. Furthermore, image reconstruction based on SWT tends to highlight edge features, and enhances the resolution of edge details. In particular, for noisy CBCT images, we can extract more accurate gradient information from the images, thereby the accuracy of the registration can be improved.

## Abbreviations

CBCT: Cone beam-CT
IGRT: Image guided radiotherapy technology
SWT: Stationary wavelet transform
NMI: Normalized mutual information
3D CRT: Three-dimensional conformal radiotherapy
IMRT: Intensity modulated radiation therapy
MI: Mutual information
